# Pathophysiology of Endometriosis: Role of High Mobility Group Box-1 and Toll-Like Receptor 4 Developing Inflammation in Endometrium

**DOI:** 10.1371/journal.pone.0148165

**Published:** 2016-02-12

**Authors:** Bo Hyon Yun, Seung Joo Chon, Young Sik Choi, SiHyun Cho, Byung Seok Lee, Seok Kyo Seo

**Affiliations:** 1 Department of Obstetrics and Gynecology, Severance Hospital, Yonsei University College of Medicine, Seoul, Republic of Korea; 2 Institute of Women’s Life Medical Science, Yonsei University College of Medicine, Seoul, Republic of Korea; 3 Department of Obstetrics and Gynecology, Gil Hospital, Graduate School of Medicine, Gachon University of Medicine and Science, Inchon, Republic of Korea; 4 Department of Obstetrics and Gynecology, Gangnam Severance Hospital, Yonsei University College of Medicine, Seoul, Republic of Korea; University of Aberdeen, UNITED KINGDOM

## Abstract

Oxidative stress has been proposed as a potential factor associated with the establishment and progression of endometriosis. Although a few studies have shown possible mechanisms which may play roles in development, progression of endometriosis, few are known in regards of initiation of the disease, especially in the relationship with endometrium. The aim of our study was to investigate whether normal endometrium may be changed by Damage-associated molecular patterns (DAMPs), which may contribute developing pathologic endometrium to induce endometriosis. Endometrial tissues were obtained from 10 patients with fibroids undergoing hysterectomy at a university hospital. High mobility group box-1 (HMGB-1), which is a representative DAMP, has been chosen that may induce alteration in endometrium. In preceding immunohistochemistry experiments using paraffin-block sections from endometriosis (N = 33) and control (N = 27) group, retrospectively, HMGB-1 expression was shown in both epithelial and stromal cell. HMGB-1 expression was significantly increased in secretory phase of endometriosis group, comparing to the controls. To examine the alteration of endometrial stromal cell (HESC) by oxidative stress in terms of HMGB-1, cell proliferation and expression of its receptor, TLR4 was measured according to recombinant HMGB-1 use. Cell proliferation was assessed by CCK-8 assay; real-time PCR and western blotting were used to quantify Toll like receptor 4 (TLR4) mRNA and protein expression respectively. A TLR4 antagonist (LPS-RS) and an inhibitor of the NF-κB pathway (TPCA-1, an IKK-2 inhibitor) were used to confirm the relationships between HMGB-1, TLR4, and the NF-κB pathway. Passive release of HMGB-1 was significantly proportional to the increase in cell death (P<0.05). HESCs showed significant proliferation following treatment with rHMGB-1 (P<0.05), and increased TLR4 expression was observed following rHMGB-1 treatment (P<0.05) in a concentration-dependent manner. Treatment with a TLR4 antagonist and an NF-κB inhibitor resulted in suppression of rHMGB-1-induced HESC proliferation (P<0.05). Levels of IL-6 were significantly decreased following treatment with an NF-κB inhibitor (P<0.05). Our results support the development of altered, pathological endometrium resulted from oxidative stress in normal endometrium. These findings may provide important insights into the changes in endometrium linking the development and progression of endometriosis.

## Introduction

Endometriosis is a gynecological disorder that causes pelvic pain and infertility in women of reproductive age [1]. While the etiology of the disease remains unclear, retrograde menstruation, coelomic metaplasia, and lymphovascular metastasis have been shown to be the major pathological characteristics of endometriosis. However, none of these theories can fully explain the pathogenesis of endometriosis. Because retrograde menstruation occurs in about 80% of women, while endometriosis occurs in only 10%–15% of women, additional mechanisms must contribute to the survival of ectopic endometrium outside the uterus [2].

Oxidative stress has been proposed as a potential factor associated with the establishment and progression of endometriosis [3,4]. Previous studies have reported that the levels of oxidative stress and antioxidant biomarkers found in peritoneal fluid are significantly different between patients with and without endometriosis [3]. Moreover, oxidative stress in the pelvic cavity of patients with endometriosis may be an important facilitator or inducer of chronic nuclear factor-kappa B (NF-κB) activation, enhancing NF-κB-mediated inflammatory reactions and endometriotic cell survival and growth [4]. Therefore, the vulnerability of the endometrial cells to oxidative stress and the subsequent activation of the oxidative stress-NF-κB axis may constitute the basis for the pathophysiology of endometriosis.

Damage-associated molecular patterns (DAMPs) are endogenous molecules that can initiate and perpetuate the immune response in noninfectious inflammatory response [5]. High mobility group box-1 (HMGB-1) is a representative DAMP that is localized in the nucleus of all mammalian cells [6], where it binds to DNA, stabilizes the structure of DNA, and controls transcriptional activity [7]. However, HMGB-1 may also be released into the extracellular space either actively by inflammatory cells or passively by necrosis, leading to inflammation [8]. Passively released HMGB-1 binds to receptors such as Toll-like receptor 4 (TLR4) with high affinity, and binding of HMGB-1 to TLR4 can activate NF-κB light chain, which play important roles in tumor growth and progression [9–12]. However, despite these interesting roles of HMGB-1 in the pathogenesis of various diseases, including sepsis[8], arthritis[13], ischemic injury[14], researchers are yet to study the involvement of HMGB-1 in endometriosis.

The purpose of this study was to determine whether normal endometrium may be changed by HMGB-1, acquiring increased cell proliferation and decreased apoptosis. Additionally, we further investigated whether TLR4 plays an important role in regulating inflammatory responses by NF-κB pathway in endometrial cells.

## Materials and Methods

### Participants

From March 2012 to March 2014, total 70 patients who underwent hysterectomies at Severance Hospital, Yonsei University College of Medicine were enrolled in this study. Among the participants, 60 patients were enrolled retrospectively based on their final diagnosis and were divided into the endometriosis (33 patients) and control (27 patients) groups; the histopathological slides of endometrial tissues were used for immunohistochemistry. The final diagnosis of endometriosis was made based on either pathological confirmation of ectopic endometriotic tissue or operative findings. In the control group, 27 patients had leiomyoma of the uterus, two had cervical cancer, and one had endometrial stromal nodules. For cell culture, endometrial specimens were obtained from 10 patients without endometriosis following hysterectomy for uterine fibroids. All 70 participants were in the reproductive age and had reported having regular menstrual cycles every 28–30 days. None of the participants received any hormonal treatment for at least 3 months preceding the surgery. The study was approved by the institutional review board of Severance hospital, Yonsei University College of Medicine (4-2014-0560). 10 participants who provided endometrial tissue for the cell culture were given written informed consent.

### Sample collection

Paraffin-embedded sections of endometrium were provided for immunohistochemistry. The menstrual cycle phase of the endometrium samples used for immunohistochemistry was characterized by morphological evaluation following the criteria described in a previous publication[15]: early proliferative (EP), late proliferative (LP), early-secretory (ES), mid-secretory (MS), late-secretory (LS), and menstrual (M) phases. For each phase, five tissue samples were included from the endometriosis group, and same for the control group. Endometrial tissue was obtained by curettage from all of the 10 participants at the beginning of the hysterectomy process in the operation room. The specimens were transferred into a buffered saline solution directly after acquisition and immediately sent to the laboratory for analysis.

### Study design

The experiments were designed to confirm dynamic expression of HMGB-1 and TLR4 in endometrium using immunohistochemistry, since there are few data reporting the expression of HMGB-1 in human endometrium. Separately, endometrial stromal cell culture was performed using endometrial tissue from 10 participants without endometriosis, to examine the normal stromal cell response to rHMGB-1 and various treatment, in terms of cell proliferation and TLR4 expression. To examine the passive release of HMGB-1 by necrosis in the endometrium, HMGB-1 was examined after inducing cell necrosis by H_2_O_2_. Cell death was measured using 3-(4, 5-dimethylthiazol-2-yl)-2, 5-diphenyltetrazolium bromide (MTT) assays, and the levels of HMGB-1 were quantified by enzyme-linked immunosorbent assays (ELISAs) and western blots. To determine the relationships of HMGB-1 and TLR4 expression with endometrial proliferation, cultured endometrial cells were treated with recombinant HMGB-1 (rHMGB-1), which was followed by cell proliferation assay and quantitative real time PCR (qRT-PCR), western blot for TLR4 RNA and protein expression. To confirm the association of HMGB-1 and TLR4 in NF-kB pathway, cell proliferation and TLR4 expression were examined applying TLR4 antagonist (LPS-RS) and inhibitor of NF-kB pathway (TPCA-1, IKK-β inhibitor), adding to rHMGB-1 treatment respectively. In experiment inhibiting NF-kB pathway with TPCA-1, level of IL-6 was measured to confirm the inhibition and compare with the appearance of TLR4 expression.

### Immunohistochemistry

Paraffin-embedded specimens of endometrium from endometriosis and control groups, were sectioned at 7μm, prepared with xylene and ethanol, pressurized and heated with proteinase K (Welgene, Daegu, Korea) for antigen retrieval. Endogenous peroxidase activity was quenched with 3% H_2_O_2_ for 10 minutes and washed in buffered saline solution (PBS; Welgene, Daegu, Korea) for 5 minutes twice. Unspecified binding of the first antibody was blocked by 1 hour incubation step in 5% bovine serum albumin (BSA; Gibco, Invitrogen, Carlsbad, CA, USA). Slides were incubated in a humidified chamber overnight at 4°C with HMGB-1 rabbit polyclonal antibody at 1:100 dilution (ProteinTech, Chicago, IL, USA). Tissue sections were washed with PBS, tris-buffered saline (TBS; Gibco, Invitrogen) was used for antibody dilutions and washes. Sections were incubated with rabbit polyclonal secondary antibodies (Vector Laboratories, Burlingame, CA, USA) for 20 min at room temperature. Diaminobenzidine was used as a chromogen at 1 mg/mL, with 0.3% H_2_O_2_ as a substrate. All sections were counterstained with hematoxylin for 1 min and washed with tap water. After dehydration, mounting was performed. Slides were documented using an Olympus BX 40F-3 microscope (Olympus, Tokyo, Japan). Intensities of immunoreactive precipitates were scored in epithelial and stromal cell compartments using Olympus DP controller software.

### Interpretation of immunohistochemical staining

The immunohistochemical histological score (H score) was used to show the immunoreactivity in stained areas of the endometrial glands and stroma, semiquantitatively. The H-score was calculated by the formula [Hscore¼ *P*(*Pi* x *i*)/100], where *Pi* stands for the percentage of positive cells for each intensity and *i* stands for the range of staining intensity. *Pi* was assigned as follows: 0 for <5%, 1 for 5% to 25%, 2 for 25% to 50%, 3 for 50% to 75%, and 4 for >75%. The staining intensity (*i*) was assigned using a 4-point scale as follows: 0 ¼ negative, 1 ¼ weak, 2 ¼ moderate, and 3 ¼ strong. At least 3 randomly chosen areas from each sample were evaluated and averaged for each compartment. The scoring procedure was carried out by 2 different, blinded reviewers without any information about the clinical background.

### Cell culture

Human endometrial stromal cells (HESCs) were obtained from tissues of 10 participants, after washing and sectioning in PBS, followed by treatment with 0.25% trypsin/EDTA (Gibco, Invitrogen) for 30 min at 37°C. During the incubation, cells were tapping every 5 min to facilitate dissociation. Supernatants were removed by centrifugation at 2000 rpm for 5 min, and HESCs were resuspended in Dulbecco’s modified Eagle’s medium/F12 (DMEM/F12, HyClone, Logan, UT, USA) supplemented with 10% fetal bovine serum (FBS; Gibco, Invitrogen) and 2% penicillin-streptomycin (P/S; Gibco, Life Technologies, Grand Island, NY, USA). After 1 min, 1 × 10^6^ cells were plated in DMEM/F12 and incubated at 37°C in a 5% CO_2_ incubator for 24 h.

### Measurement of passive HMGB-1 release after induction of necrosis

For measurement of passive HMGB-1 release, 3 × 10^5^ endometrial cells were plated in 6-well plates. After 24 h of incubation at 37°C, the medium was changed to serum-free DMEM/F12. Endometrial cells were then incubated with 0, 25, or 50 μM H_2_O_2_ for 1 h. Levels of HMBG-1 in the supernatants were then analyzed by ELISA and western blotting.

### MTT assays

MTT solution (100 μL, Sigma) was added to each well, and culture plates were incubated at 37°C for 4 h in a 5% CO_2_ incubator. The supernatants were harvested for subsequent ELISA and western blot analysis. After removing medium, 500 μL dimethyl sulfoxide (DMSO; Sigma) was added to each well, and plates were then incubated for 10 min on a shaker. Finally, the optical density (OD) was measured at 562 nm with a VersaMax microplate reader (Molecular Devices, Sunnyvale, CA, USA).

### ELISA

After harvesting supernatants, HMGB-1 was quantified using an HMBG-1 ELISA kit (Shino-Test Corporation, Kanagawa, Japan), according to the manufacturer’s protocol. The OD was measured at 450 nm with a VersaMax microplate reader (Molecular Devices). Measurement of IL-6 (BD Bioscience, San Jose, CA, USA) was performed as a control.

### Western blotting

Cells were lysed using radioimmunoprecipitation assay buffer (RIPA buffer; Intron, iNtRON Biotechnology, Sungnam, Korea) containing protease inhibitor cocktail (Cell Signaling Technology, Beverly, MA, USA), mixed, and divided in 40-μL aliquots. The lysates were collected and centrifuged at 13000 rpm at 4°C for 30 min, and protein levels in the supernatants were quantified using a BCA protein assay kit (Thermo Scientific, Hudson, NH, USA). Western blotting was then performed using the harvested supernatants. Thirty micrograms of each lysate was boiled in 5×buffer, and supernatants were separated by sodium dodecyl sulfate polyacrylamide gel electrophoresis (SDS-PAGE) on 8% gels and transferred to polyvinylidene fluoride (PVDF) membranes (Millipore, Eschborn, Germany). After blocking with BSA at room temperature for 1 h, the membranes were incubated with primary antibodies specific for HMGB-1 (monoclonal anti-rabbit, 1:50000; Abcam, Cambridge, MA, USA), TLR4 (polyclonal anti-rabbit, 0.3 μg/mL, GeneTex, Irvine, CA, USA), and GAPDH (monoclonal anti-mouse, 0.1 μg/mL, Millipore) overnight at 4°C. The membranes were then incubated with anti-mouse antibodies (IgG, 0.27 μg/mL, Jackson, West Grove, PA, USA) and anti-rabbit antibodies (IgG, 0.27 μg/mL, Jackson) conjugated with horseradish peroxidase (HRP) for 1 h at room temperature. Detection was facilitated by enhanced chemiluminescence (ECL) solution (Advansta, San Francisco, CA, USA), and the bands were quantified by densitometry using Image J software.

### Cell treatment and proliferation assays (CCK-8 assays)

Endometrial cells were seeded in 6-well tissue culture plates at a density of 1 × 10^5^ cells per well. The culture medium was changed to DMEM/F12 with 2% FBS after 24 h of incubation. After changing the media, endometrial cells were treated with rHMGB-1 (Sino, Beijing, China) at a concentration of 15 ng/mL for 12, 24, or 48 h or treated with rHMGB-1 at concentrations of 0, 5, 10, or 15 ng/mL for 48 h. For suppression of TLR4, endometrial cells were treated with 100 ng/mL LPS-RS (InvivoGen, San Diego, CA, USA) for 30 min. For suppression of NF-κB, endometrial cells were treated with 2 μM TPCA-1 (Cayman, Ann Arbor, MI, USA) for 1 h. Both treatments were followed by rHMGB-1 treatment at concentrations of 0, 5, 10, or 15 ng/mL for 48 h. Thereafter, 100 μL of CCK-8 (Cell Counting Kit-8; Dojindo, Japan) was added to each well, and plates were incubated at 37°C for 1 h. Supernatants were transferred to 96-well plates after incubation, and the OD was measured at 450 nm using a VersaMax microplate reader (Molecular Devices) to measure cell proliferation rates.

### RNA isolation and qRT-PCR

Cell-free total RNA was extracted from cell lysates using an RNeasy mini kit (Qiagen, Hilden, Germany). cDNA was synthesized from 1 μg total RNA using oligo-dT primers (Invitrogen). qRT-PCR for TLR4 was carried out using an ABI StepOnePlus (Applied Biosystems, Foster City, CA, USA) and SYBR green RT PCR master mix (Toyobo, Osaka, Japan). The primers used for TLR4 were as follows: 5′-CAGAGTTTCCTGCAATGGATCA-3′ (sense) and 5′-GCTTATCTGAAGGTGTTGCACAT-3′ (antisense), and the primers used for glyceraldehyde-3-phosphate dehydrogenase (GAPDH), which was used as an endogenous reference gene, were as follows: 5′-TCGACAGTCAGCCGCATCTTCTTT-3′ (sense) and 5′-ACCAAATCCGTTGACTCCGACCTT-3′ (antisense). The PCR conditions were as follows: 95°C for 10 min, followed by 40 cycles of 95°C for 15 s, 60°C for 1 min, and 95°C for 15 s. Data were normalized to the expression of GAPDH, and PCR products were separated on a 1.5% agarose gel containing ethidium bromide, for quantification by densitometry using Image J software (National Institutes of Health, USA).

### Proinflammatory cytokine measurement

After HESC treating TPCA-1 with rHMGB-1, 2μM of TPCA-1 (Caymen, Ann Arbor, MI, USA) for 30 minutes, followed by rHMGB-1 treatment with 0, 5, 10, 15ng/mL for 48 hours, the supernatant was harvested for ELISA. IL-6 was quantified using IL-6 ELISA kit (BD bioscience, San Jose, CA, USA), according to the manufactures’ protocol. The OD was measured at 450nm with and ELISA Reader.

### Statistical analysis

To determine the significance of difference in the levels of mRNA and protein, Kruskal-Wallis tests with Dunn’s procedure for multiple comparisons were performed. Statistical analyses were performed using SPSS 20.0 (IBM, NY, USA). Differences with *P* values of less than 0.05 were considered statistically significant.

## Results

### Immunohistochemistry

**Fig 1:**
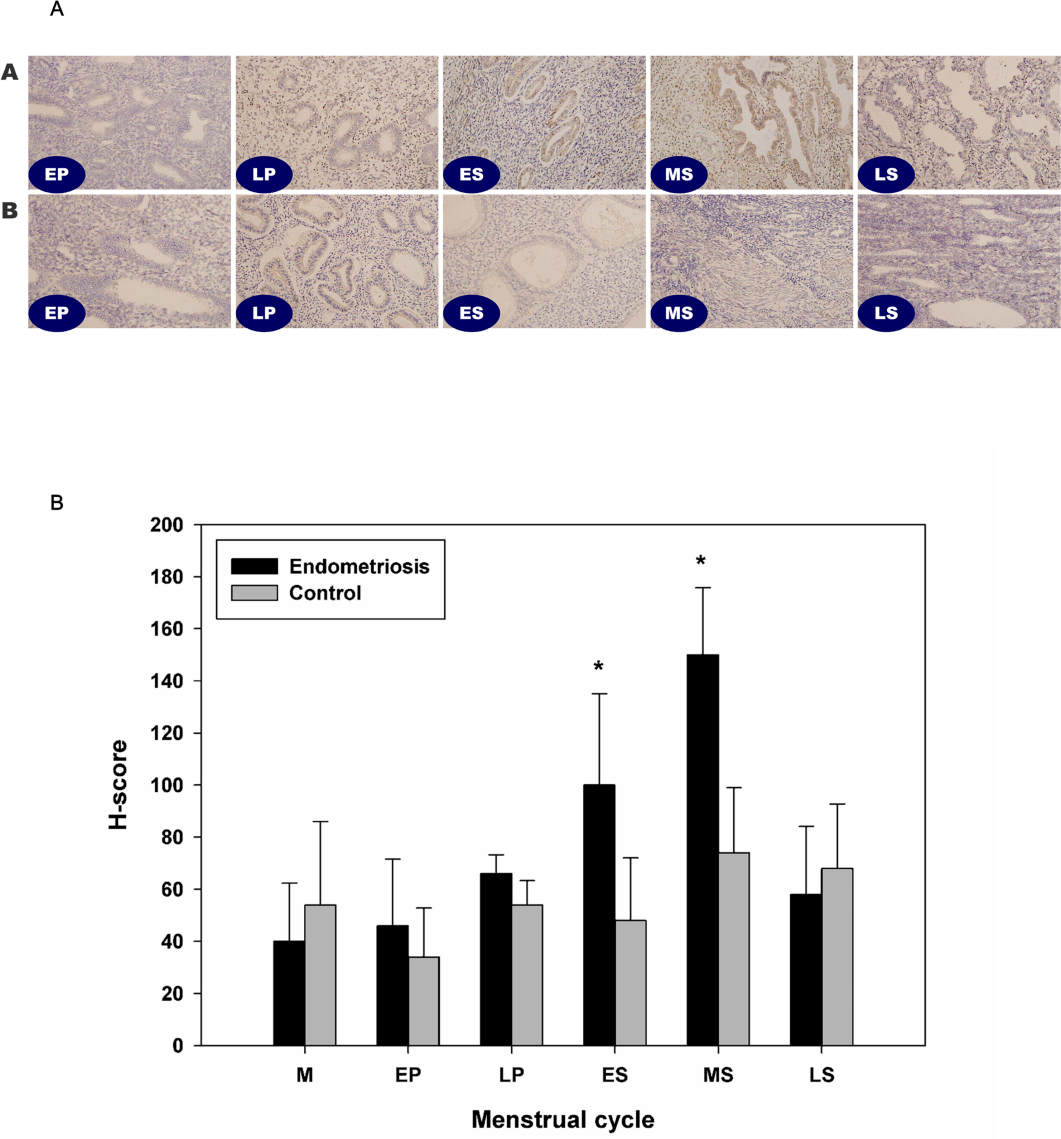
Immunohistochemistry analysis of HMGB-1 expression in the human endometrium. In control endometrium, HMGB-1 was expressed in the glandular cells of the epithelium and in stromal cells (N = 27). No significant differences were observed according to the menstrual cycle (A). In EM from a patient with endometriosis, significantly increased HMGB-1 expression was detected in the early and mid-secretory phase as compared to the control EM (N = 33, B). There were no significant differences in HMGB-1 expression between the two groups during the proliferative phase. *, *P* < 0.05 versus the control.

On immunohistochemistry, the results showed that no significant difference in expression according to the menstrual cycle, in the control group (Fig 1). HMGB-1 was expressed in both epithelial and stromal cell in control endometrium. Comparing to the controls, significantly increased expression of HMGB-1 was shown at early and mid-secretory phase in patients with endometriosis.

### Passive release of HMGB-1 after induction of necrosis in HESCs

**Fig 2:**
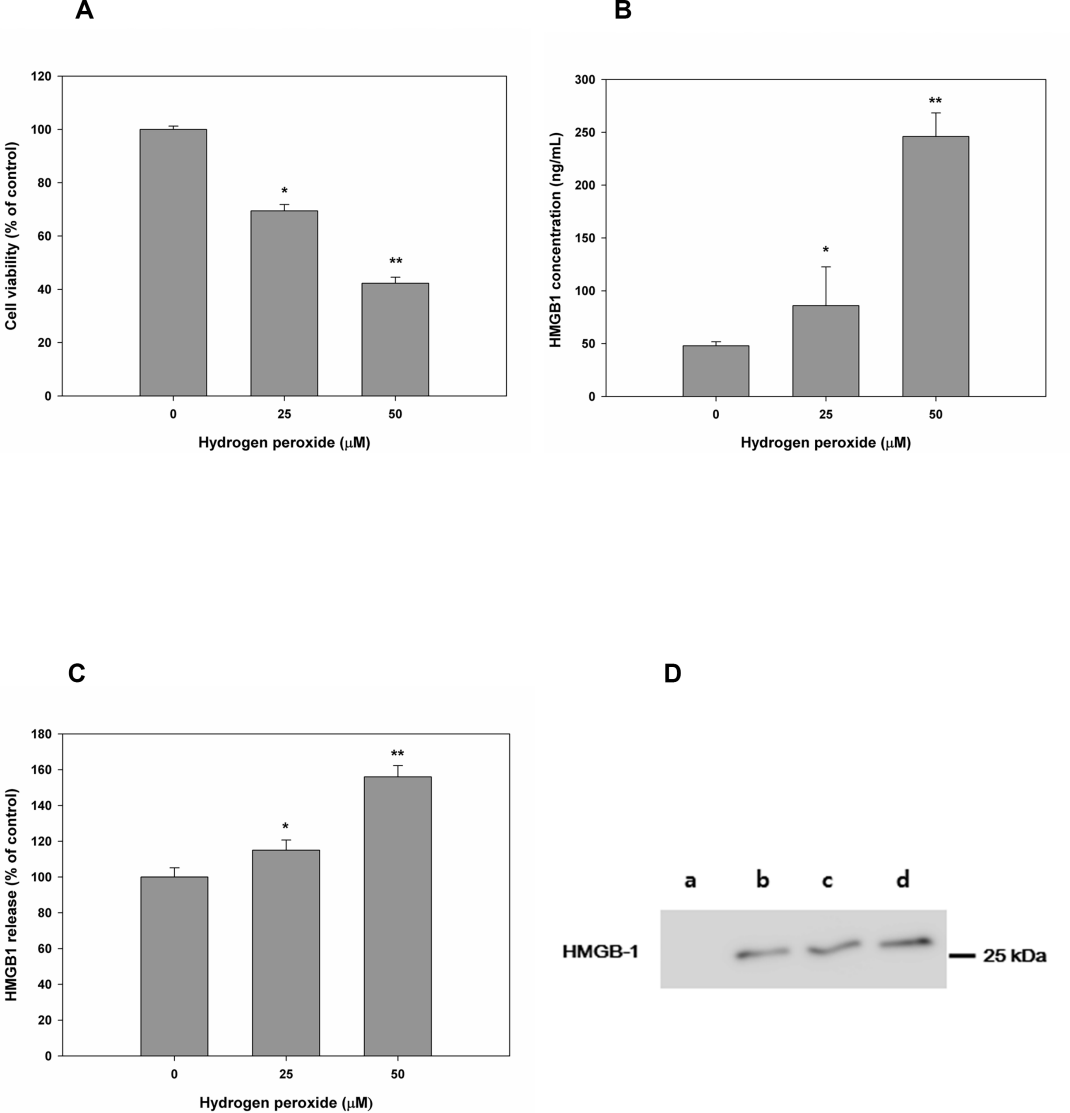
Induction of necrosis in HESCs altered the secretion of HMGB-1. Cell viability was detected by MTT assays after treatment of HESCs with H_2_O_2_ (A). Extracellular release of HMGB-1 was measured following H_2_O_2_-induced necrosis. Relative mRNA (B) and protein levels (C, D) are shown. *, *P* < 0.05 versus 0 μM H_2_O_2_; **, *P* < 0.05 versus 25 μM H_2_O_2_. a, fresh medium was used as a control; b, 0 μM H_2_O_2_; c, 25 μM H_2_O_2_; d, 50 μM H_2_O_2_.

To confirm the passive secretion of HMGB-1 induced by cell necrosis, we treated HESCs with 0, 25, or 50 μM H_2_O_2_ (Fig 2). MTT assays showed that there was a gradual decrease in cell viability depending on the concentration of H_2_O_2_ (Fig 2A). In contrast, secretion of HMGB-1 was significantly increased in proportion to the concentration of H_2_O_2_; HMGB-1 48.00±37.11ng/mL for 0 μM H_2_O_2_, 79.55 ± 36.66ng/mL for 25μM H_2_O_2_, and 240.00± 22.26ng/mL for 50μM H_2_O_2_, respectively (Fig 2B–2D).

### Effects of rHMGB-1 on cell proliferation and TLR4 expression

**Fig 3:**
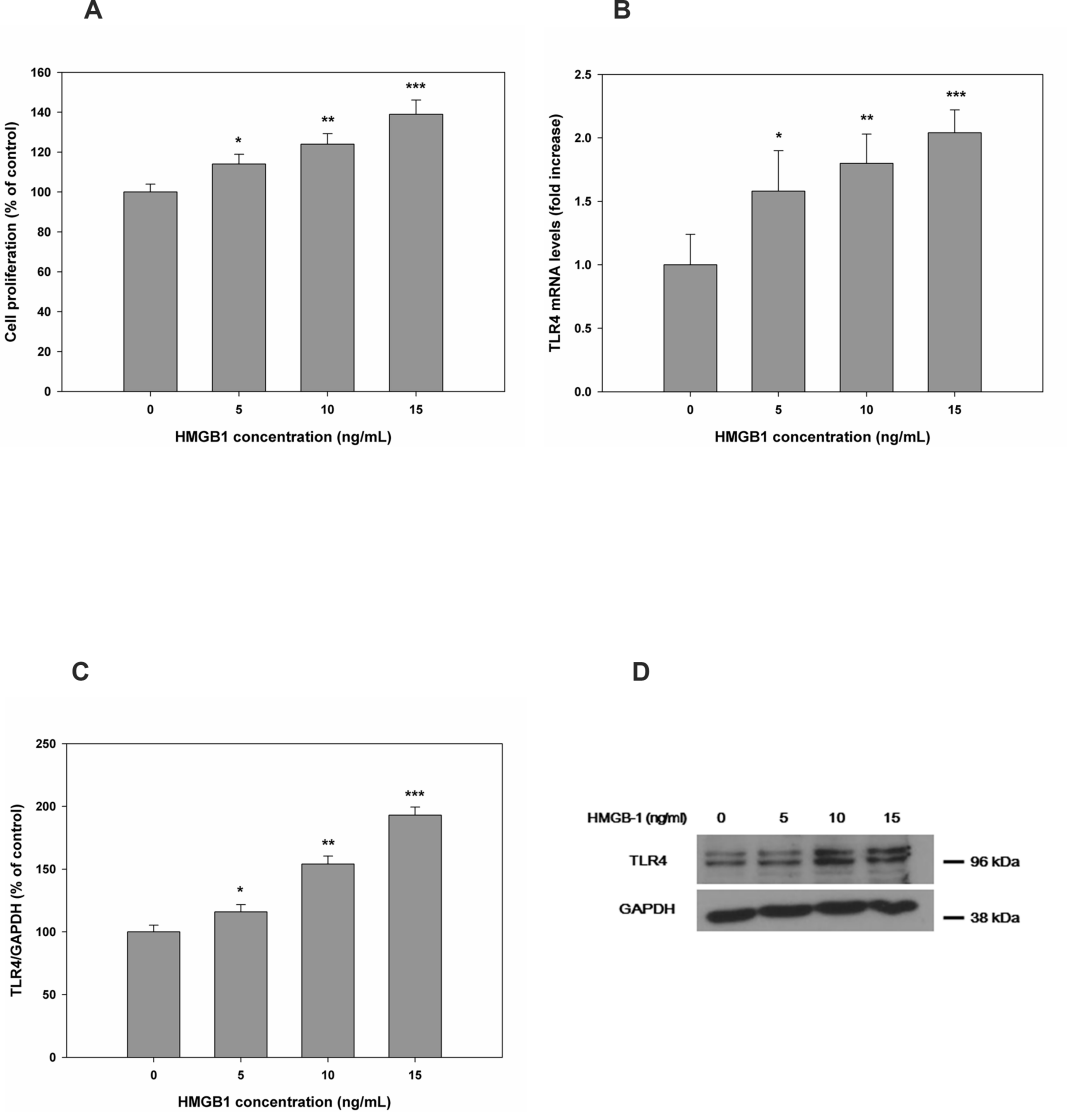
HESC proliferation and TLR4 expression were altered in a concentration-dependent manner after treatment with rHMGB-1. At 48 h after treatment with different concentrations of rHMGB-1, cell proliferation was measured (A). Cell proliferation after 24 or 48 h of treatment with rHMGB-1 (B). Relative mRNA and protein levels of TLR4 are shown following treatment with rHMGB-1 (C, D). *, *P* < 0.05 versus the control; **, *P* < 0.05 versus 5 ng/mL rHMGB-1; ***, *P* < 0.05 versus 10 ng/mL rHMGB-1.

**Fig 4:**
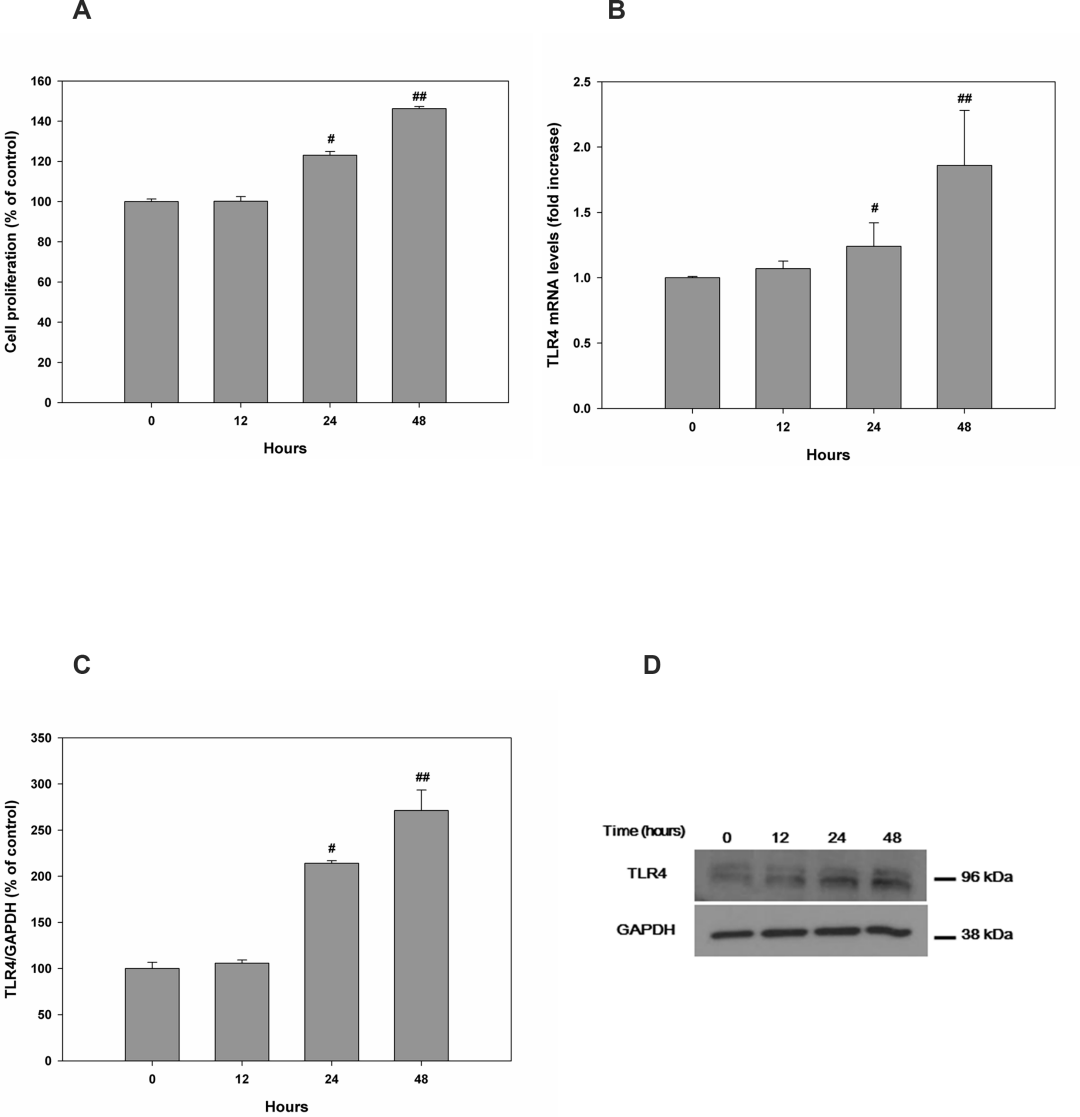
HESC proliferation and TLR4 expression were altered in a time-dependent manner after treatment with rHMGB-1. Changes in cell proliferation were measured at 0, 12, 24, and 48 h after treatment with 15 ng/mL rHMGB-1 (A). Relative mRNA (B) and protein levels (C, D) of TLR4 are shown. #, *P* < 0.05 versus 12 h; ##, *P* < 0.05 versus 24 h.

Following treatment of HESCs with different concentrations of rHMGB-1 for 0–48 h, cell proliferation was significantly increased in a concentration- (Fig 3 and S1 Fig) and time-dependent manner (Fig 4 and S1 Fig) as compared with that observed in the control. rHMGB-1 also caused a concentration- and time-dependent increase in TLR4 mRNA and protein expression. TLR4 protein expression showed 100.0±2.97% for 0 ng/mL of HMGB-1, 127.63±4.72% for 5 ng/mL of HMGB-1, 208.44±8.09% for 10 ng/mL of HMGB-1, and 296.83±15.20% for 0 ng/mL of HMGB-1, compared to the GAPDH control, respectively (Fig 3C and 3D). Time-dependent manner TLR4 protein increase at treatment of rHMGB-1 showed 100.0±6.73% for 0 hour of HMGB-1, 105.84±3.5% for 12 hours of HMGB-1, 213.88±2.90% 24 hours of HMGB-1, and 271.18±22.35% for 48 hours of HMGB-1 treatment, compared to the GAPDH control, respectively (Fig 4C and 4D).

### Effects of combined rHMGB-1 treatment and TLR4 inhibition on cell proliferation and TLR4 expression

**Fig 5:**
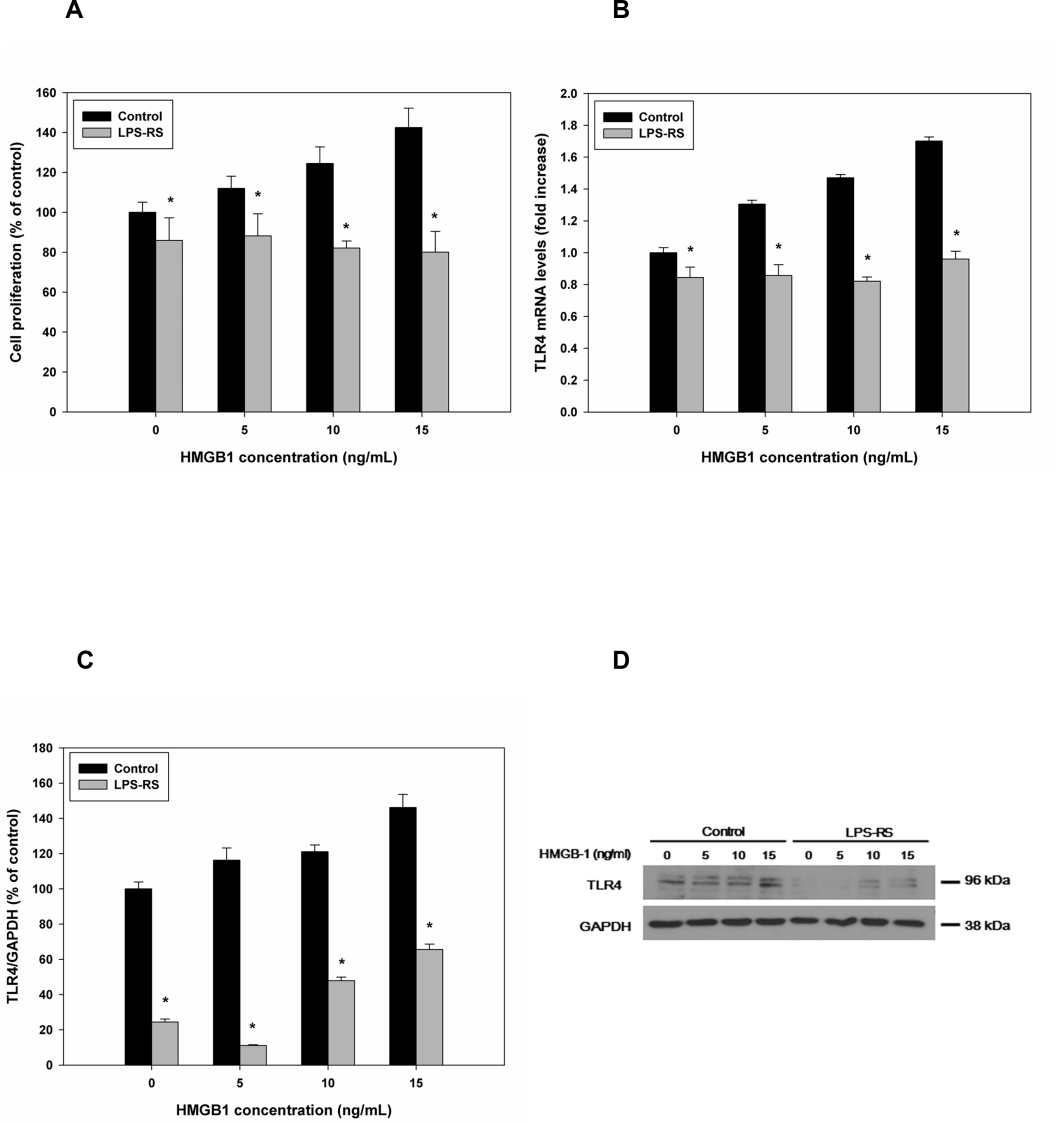
Effects of TLR4 inhibition on HESC proliferation and TLR4 expression following treatment with rHMGB-1. Cells were treated with the TLR4 antagonist LPS-RS, rHMGB-1-induced cell proliferation was measured (A). TLR4 mRNA and protein levels were measured (B–D). *, *P* < 0.05 versus the control.

Treatment of HESCs with LPS-RS and rHMGB-1 markedly blocked HESC proliferation compared to the control, although no significant differences were observed among different concentrations of rHMGB-1 (S2 Fig and Fig 5A). The combined treatment blocked the rHMGB-1-induced increases in TLR4 expression, and significantly decreased TLR4 mRNA and protein levels were observed compared with the control cells at each concentration of rHMGB-1 (Fig 5B–5D). The expression of TLR4 protein according to the LPS-RS and rHMGB-1 treatment was 24.45±1.68% for 0 ng/mL of HMGB-1, 11.08±0.49% for 5 ng/mL of HMGB-1, 47.94±1.95% for 10 ng/mL of HMGB-1, 65.57±3.30% for 15 ng/mL of HMGB-1 compared to the GAPDH control, respectively (Fig 5C and 5D).

### Effects of combined rHMGB-1 treatment and NF-κB inhibition on cell proliferation and TLR4 expression

**Fig 6:**
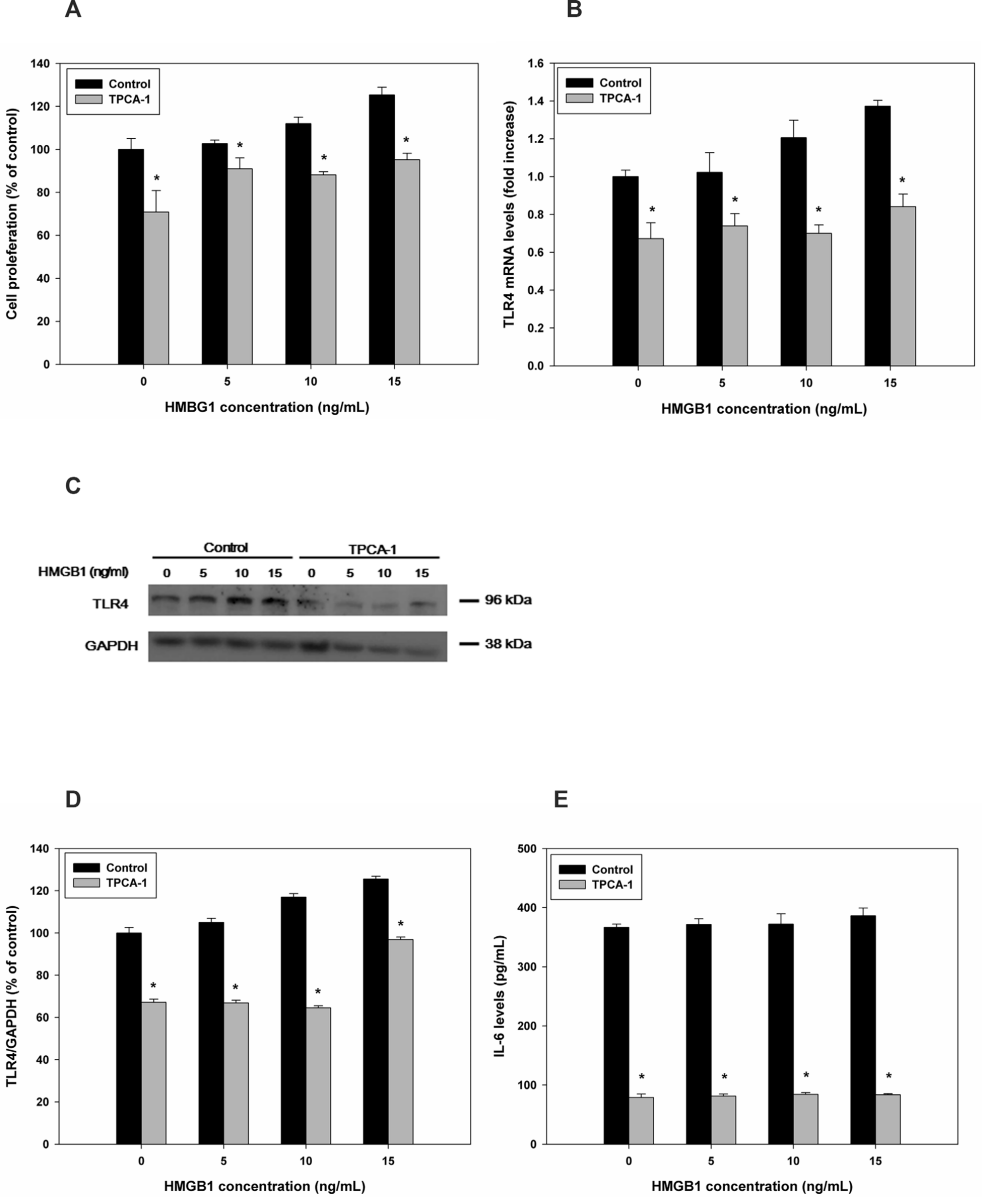
Effects of NF-κB inhibition on rHMGB-1-induced HESC proliferation and TLR4 expression. The NF-κB pathways was blocked using TPCA-1, and rHMGB-1-dependent cell proliferation was measured (A). TLR4 expression was analyzed using qRT-PCR (B) and western blotting (C, D) following treatment with different concentrations of rHMGB-1. Changes in IL-6 secretion were measured following inhibition of the NF-κB pathway by TPCA-1 (E). *, *P* < 0.05 versus the control.

Next, we analyzed the effects of NF-κB inhibition on cell proliferation and TLR4 expression. HESCs were treated with TPCA-1 combined with rHMGB-1, and cell proliferation was significantly blocked compared with the control (S3 Fig and Fig 6A), similar to the results of TLR4 inhibition. After treatment with TPCA-1 prior to rHMGB-1 in endometrial cells, TLR4 mRNA and protein levels were significantly decreased compared with that in the control (Fig 6B–6D), with no concentration-dependent effects observed for rHMGB-1. The protein expression of TLR4 compared to the control (GAPDH) was shown 67.18±0.50% for 0ng/mL of HMGB-1, 66.90±0.29% for 5ng/mL of HMGB-1, 64.57±0.03% for 10 ng/mL of HMGB-1, and 106.87±0.18% for 15ng/mL of HMGB-1, respectively. Furthermore, the secretion of IL-6, a pro-inflammatory cytokine that is known to be the end product of NF-κB pathway activation, was significantly decreased after treatment with TPCA-1 (Fig 6E).

## Discussion

In this study, we sought to elucidate the mechanisms mediating development of pathological endometrium by oxidative stress in endometriosis. Immunohistochemistry showed presence of HMGB-1 in human endometrial cells and significantly increased HMGB-1 expression during secretory phase in endometriosis group compared to the normal controls, additionally. We found that passively released extracellular HMGB-1 bound to TLR4 and may induce sterile inflammation via a cascade involving the NF-κB pathway in human endometrium. The effects of HMGB-1 treatment were blocked by both TLR4 inhibition and NF-κB inhibition, demonstrating possible involvement of the NF-κB pathway in this mechanism. Based on this, we developed a model in which DAMPs cooperate in the inflammatory reaction in the HESC by responding to oxidative stress, which may account for modified ectopic endometrium in pelvic cavity that lead to endometriosis.

Many studies that were conducted in regards to pathogenesis of endometriosis have been made various suggestions [16–19]. As well known, the origin of ectopic endometrium is not agreed in Sampson’s implantation theory, since it does not explain fully in aspects of extra-pelvic endometriosis. However, at least in pelvic endometriosis, retrograde menstruation and implantation on the peritoneal surface leave little for doubt. The mechanism that may contribute successful ectopic implantation has been suggested as one or two: either or both, molecular defect and immunological abnormalities [20]. The implanted endometrium may proceed to induce chronic inflammation by time past, adding burden of iron [21]. The factors that are related to the progress of the disease are suggested in variety such as epigenetic changes in endometriotic tissue, decrease in apoptotic factors, increase in angiogenic factors and cytokines that resulted in stromal-cell defect and progesterone resistance [16,22]. Although different character of ectopic endometrium (compared to the eutopic endometrium) has been suggested, the limitation of it may be that unknown origin of the difference. To say, it is not clear where the alteration has been occurred–whether the eutopic endometrium has been altered or after retrograde menstruation, has it been altered? Moreover, the factor which contributes to the alteration is not clarified, mostly suspected that immunologic factor may affect[17]. Our study has been started from the question, and the results suggest eutopic endometrium has a potential of alteration, under the influence of oxidative stress. We have presented a pathway for an example that may involve the alteration of the endometrium by DAMP, the alteration may be aggravated in environment with sustained oxidative stress.

Extracellular HMGB-1 is a representative DAMP that acts as an innate ligand sending signals of cell and tissue damage; other DAMPs include reactive oxygen stress (ROS), heat shock protein, fibrinogen, and adenosine triphosphate. Secretion of HMGB-1 into the extracellular space leads to direct binding of HMGB-1 to transmembrane TLR proteins and RAGEs, leading to NF-κB pathway activation [5,8]. In the pro-oxidant environment, HMGB-1 activates NF-κB by binding to TLR4 and inducing the pro-inflammatory response. This pathway has been shown to be involved in the pathogenesis of various diseases, such as rheumatoid arthritis [13], lupus erythematosus [23], atherosclerosis [24], and sepsis [8]. The pro-inflammatory activity of HMGB-1 depends on the domain B box, which is composed of two crucial binding sites for TLR4 and RAGE [25]. The pro-inflammatory activity of HMGB-1 has been shown to be dependent upon its redox state [26]. In the oxidized environment, e.g., during increased ROS production, the redox-state of HMGB-1 is necessary to maintain the redox state of the cell environment. However, the reduction in oxidized HMGB-1 by thioredoxin (TRX) is a slower reaction than other reducing reactions mediated by TRX [26]. Due to the low efficacy of the redox system, HMGB-1 may require increased TRX levels to maintain the redox state and induce the immune reaction. Indeed, previous studies from our lab have shown that the immune system is dysregulated in the eutopic endometrium of patients with endometriosis [27]. In the current study, we found that normal, eutopic endometrium may be transformed by HMGB-1, which may be released by oxidative stress. Because of the sensitivity of HMGB-1 to the oxidation-reduction state of the surrounding environment, HMGB-1-mediated NF-κB signaling may be the predominant mechanism during early inflammation of the endometrium in patients with endometriosis. Analysis of TLR4 expression has also supported that the pathological endometrium affects the development of endometriosis [28].

Additionally, although it was not the primary purpose of this study, we have performed immunohistochemistry to identify expression of HMGB-1 in endometrial samples from patients with and without endometriosis (Fig 1). With the exception of a single report of HMGB-1 expression in rat endometrium, no studies have examined the expression of HMGB-1 in endometrial tissues [29]. Immunohistochemistry analysis showed that HMGB-1 was expressed in glandular cells of the epithelium and stromal cells in control endometrium, without significant differences according to the menstrual cycle (Fig 1A). In endometrium from patients with endometriosis, significantly increased HMGB-1 expression was detected in the early and mid-secretory phase as compared to the control (Fig 1B). However, it should be stressed that the meaning of expression and secretion of HMGB-1 is completely different. In contrast to intracellular HMGB-1, which plays a physiologic role in the nucleus, extracellularly secreted HMGB-1 acts as a DAMP, resulting in induction of the inflammatory response. Because of the observed increase in expression in the early and mid-secretory phases, aberrant expression of HMGB-1 may be associated with interrupted implantation in patients with endometriosis, which may partly explain the development of progesterone resistance in women with endometriosis [30]. Further studies are needed to investigate in terms of this possibility.

Our study has shown a detailed inflammatory process through which DAMPs and receptors signal in HESCs. Our results also supported previous findings demonstrating the activation of the NF-κB pathway in the endometrium of patients with endometriosis and provided supportive evidence of the implantation theory, which suggests that the pathologic endometrium is implanted into the peritoneal cavity, leading to the development of endometriosis. However, our study was limited in that we only performed in vitro analysis using HESCs. Therefore, in vivo studies are required using eutopic and ectopic endometrium. Additionally, still remains unknown that whether the modification of endometrium initiates, inside the endometrial cavity or the pelvic cavity. However, the study gives supporting evidence that oxidative stress may be a key factor, which develops endometriosis or not in women with retrograde menstruation. Since the semiquantification of HMGB-1 in endometrium by immunohistochemistry remains as another limitation, further separated study is required.

In conclusion, our results herein support the development of altered, pathological endometrium resulted from oxidative stress in normal endometrium. These findings may provide important insights into the changes in endometrium linking the development and progression of endometriosis.

## Supporting Information

S1 Fig TLR4 expression according to rHMGB-1 treatment- original data. (PDF)

S2 Fig Effects of TLR4 inhibition on TLR4 expression following rHMGB-1 treatment–original data. (PDF)

S3 Fig Effects of NF-κB inhibition on TLR4 expression following rHMGB-1 treatment- original data. (PDF)
