# Correction: Pathophysiology of Endometriosis: Role of High Mobility Group Box-1 and Toll-Like Receptor 4 Developing Inflammation in Endometrium

**DOI:** 10.1371/journal.pone.0203741

**Published:** 2018-09-05

**Authors:** Bo Hyon Yun, Seung Joo Chon, Young Sik Choi, SiHyun Cho, Byung Seok Lee, Seok Kyo Seo

There is an error in the penultimate sentence under the subheading “Participants” in the Materials and Methods section. The correct sentence is: The study was approved by the institutional review board of Severance hospital, Yonsei University College of Medicine (4-2012-0458).
