# Supplementary material for: Pathophysiology of Endometriosis: Role of High Mobility Group Box-1 and Toll-Like Receptor 4 Developing Inflammation in Endometrium
Source: PLoS One. 2016 Feb 12;11(2):e0148165. doi: 10.1371/journal.pone.0148165 (PMC4752230; doi:10.1371/journal.pone.0148165)
Supplement: S1 Fig — (PDF) [file pone.0148165.s001.pdf]

**S1 Fig. TLR4 expression according to rHMGB-1 treatment- original data**

✓Time dependent

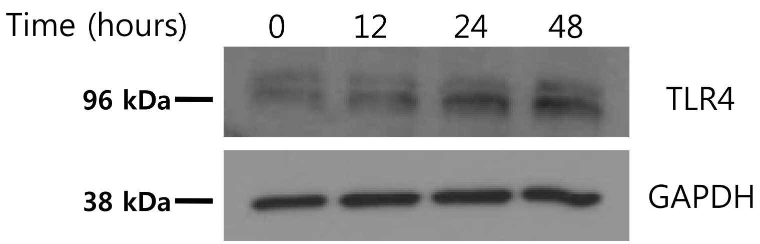


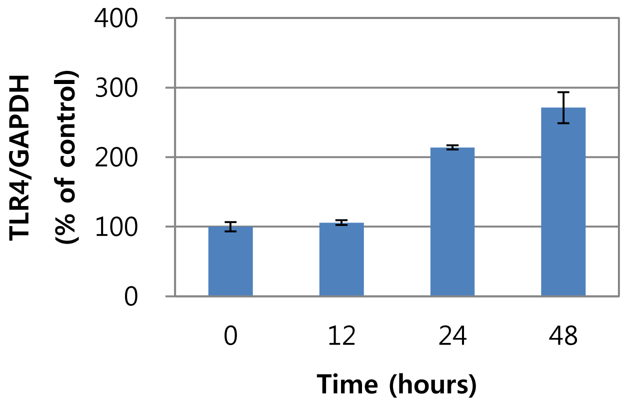


| TLR4/GAPDH | | | |
|---|---|---|---|
| hours | 1st | 2nd | 3rd |
| 0 | 1.01 | 1.06 | 0.93 |
| 12 | 1.07 | 1.09 | 1.02 |
| 24 | 2.14 | 2.11 | 2.17 |
| 48 | 2.68 | 2.50 | 2.95 |

| TLR4/GAPDH | aver | std |
|---|---|---|
| 0 | 100.00 | 6.73 |
| 12 | 105.84 | 3.50 |
| 24 | 213.88 | 2.90 |
| 48 | 271.18 | 22.35 |

| t-test | tail=1 | tail=2 |
|---|---|---|
| 0 vs. 5 | 0.137 | 0.275 |
| 5 vs. 10 | 0.000 | 0.000 |
| 10 vs. 15 | 0.023 | 0.045 |
| 1 vs. 10 | 0.000 | 0.000 |

✓Concentration dependent

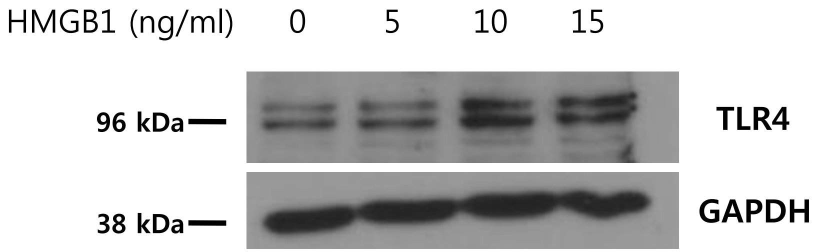


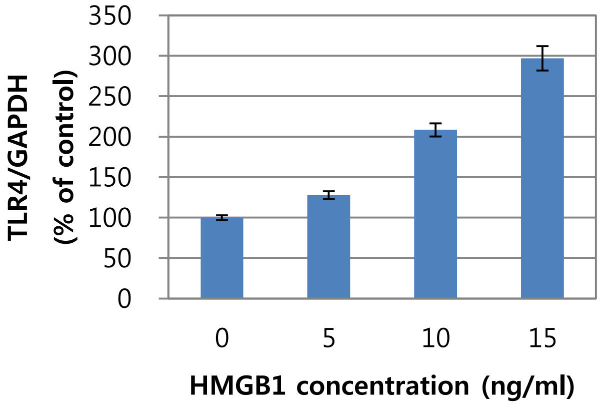


| TLR4/GAPDH | | | |
|---|---|---|---|
| | 1st | 2nd | 3rd |
| 0 | 1.00 | 0.97 | 1.03 |
| 5 | 1.28 | 1.23 | 1.32 |
| 10 | 2.10 | 2.00 | 2.16 |
| 15 | 2.99 | 2.81 | 3.11 |

| TLR4/GAPDH | aver | std |
|---|---|---|
| 0 | 100.00 | 2.97 |
| 5 | 127.63 | 4.72 |
| 10 | 208.44 | 8.09 |
| 15 | 296.83 | 15.20 |

| t-test | tail=1 | tail=2 |
|---|---|---|
| 0 vs. 5 | 0.001 | 0.002 |
| 5 vs. 10 | 0.000 | 0.000 |
| 10 vs. 15 | 0.001 | 0.003 |
| 1 vs. 10 | 0.000 | 0.001 |
