# Supplementary material for: Pathophysiology of Endometriosis: Role of High Mobility Group Box-1 and Toll-Like Receptor 4 Developing Inflammation in Endometrium
Source: PLoS One. 2016 Feb 12;11(2):e0148165. doi: 10.1371/journal.pone.0148165 (PMC4752230; doi:10.1371/journal.pone.0148165)
Supplement: S2 Fig — (PDF) [file pone.0148165.s002.pdf]

S2 Fig. Effects of TLR4 inhibition on TLR4 expression following rHMGB-1 treatment - original data

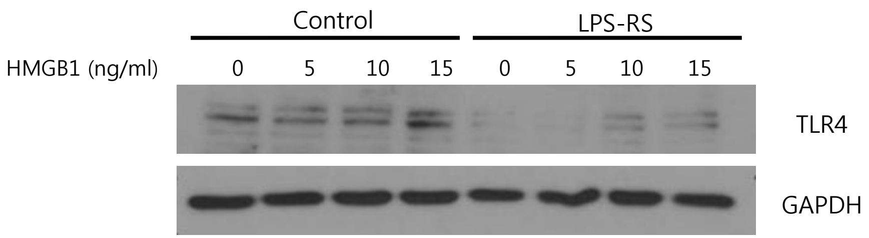

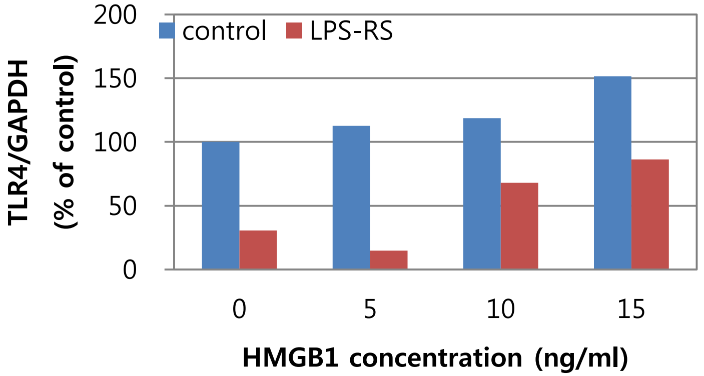

		GAPDH	TLR4	TLR4/GAPDH	% of control
control	0	22352.86	8575.326	0.383634381	100.00
	5	20133.45	8702.619	0.432246848	112.67
	10	19962.03	9079.79	0.45485297	118.56
	15	18587.5	10803.255	0.581210854	151.50
LPS-RS	0	15690.91	1837.891	0.117130923	30.53
	5	19959.69	1127.426	0.056485146	14.72
	10	18789.62	4907.083	0.26115926	68.08
	15	18784.23	6218.497	0.331048899	86.29
