# Supplementary material for: Pathophysiology of Endometriosis: Role of High Mobility Group Box-1 and Toll-Like Receptor 4 Developing Inflammation in Endometrium
Source: PLoS One. 2016 Feb 12;11(2):e0148165. doi: 10.1371/journal.pone.0148165 (PMC4752230; doi:10.1371/journal.pone.0148165)
Supplement: S3 Fig — (PDF) [file pone.0148165.s003.pdf]

S3 Fig. Effects of NF-kB inhibition on TLR4 expression following rHMGB-1 treatment - original data

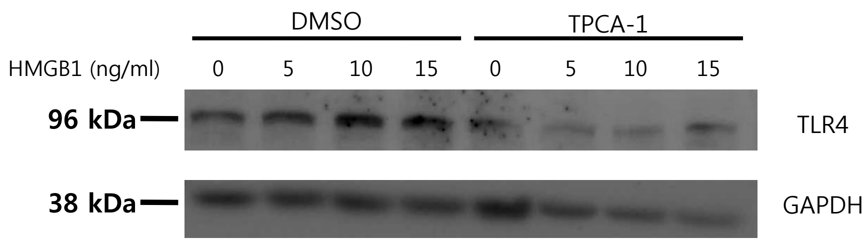

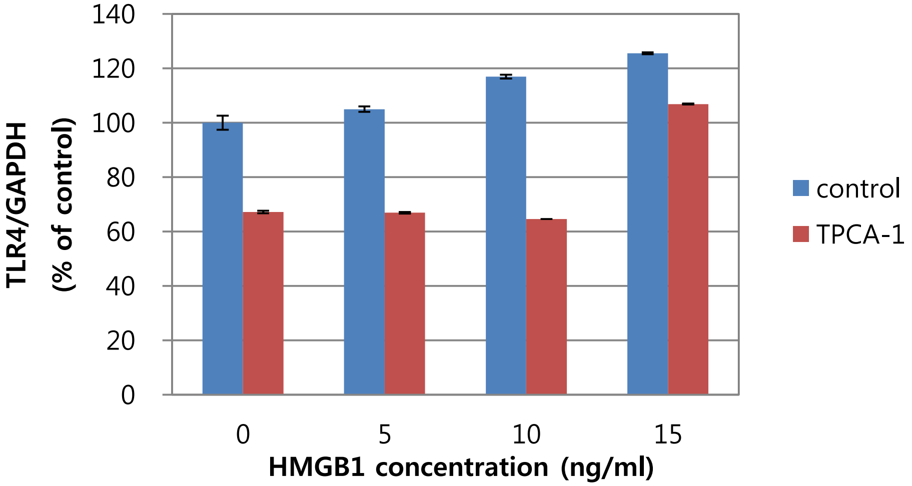

		TLR4	GAPDH	TLR4/GAPDH		% of control	std
control	0	4649.450	13346.976	0.348		100.000	2.636
	5	5008.696	13695.774	0.366		104.981	0.988
	10	6107.619	14991.676	0.407		116.950	0.672
	15	6221.057	14225.292	0.437		125.538	0.328
TPCA-1	0	4723.959	20187.512	0.234		67.175	0.495
	5	2041.541	8760.073	0.233		66.900	0.288
	10	1290.056	5734.952	0.225		64.573	0.033
	15	2135.134	5734.952	0.372		106.873	0.181
